# Do the Approved Societies Seek to Control the Doctors?

**Published:** 1922-09

**Authors:** Gordon Ward

**Affiliations:** Vice-President, Medical Practitioners' Union


### CORRESPONDENCE.

[Correspondence on all subjects is invited, but we cannot in any way be responsible for the opinions expressed by our correspondents, who must give name and address as a guarantee of good faith, but not necessarily for publication. Correspondents are reminded that conciseness greatly facilitates early insertion.]

### Do the Approved Societies Seek to Control the Doctors?

*To the Editor of* THE HOSPITAL AND HEALTH REVIEW.

SIR,—In spite of the able article by Mr. R. W. Harris in your August number, the doctors are—not without cause—very apprehensive of Approved Society control of medical benefit. The Approved Societies are in the habit of claiming that all the contributions which are paid to them (by employed, employer and the State) are properly to be regarded as funds belonging to them, and that they should have a loud voice in their expenditure. This is put by Mr. Thomas (Hansard, May 24, 1922) as follows: "We want an assurance that, in 1923, when we come to review the situation those who have paid the piper shall at least have an opportunity of calling the tune," and "We want that the Approved Societies shall themselves have a voice in determining the new arrangement, whatever it may be, between the doctors and the insured people." We doctors know that the Minister has given pledges to this effect, for we have been so told in a published interview with an official of the Ministry. Naturally we are apprehensive, naturally we can only laugh at Mr. Harris's answer to the question: "Do the Approved Societies seek to control the Doctors?" It is a commonplace of our everyday work that the Societies lose no opportunity to tighten their control.

If we look at other countries, we see many phases of this old conflict—the control of a highly specialised and technical business by persons ignorant of even its fundamentals. Before the war we had it in our own battles of the clubs; Germany (from thence came our National Health Insurance) had actual medical strikes; Austria has been worse still; our Australasian Colonies witness continual discord between the clubs and the doctors. The result of all this is bad for the public. The clubs (in our case, the Approved Societies) continually emphasise the few cases in which medical men have "gone wrong" and do their best to discredit the profession in the eyes of the people. The people recognise that if they cannot trust their doctor, there is little left on this earth which they can trust, and are accordingly made uneasy and distressed—the worst possible attitude for recovery from any ailment, and especially for recovery from the increasingly prevalent ailments of the neurasthenic type. The doctor, on the other hand, does not deny the occasional existence of ne'er-do-wells in all professions, but is conscious that the great majority of his profession are always ready to put a patient's interest before their own convenience—and this cannot be said to a like extent of any other callings, except those of a definitely religious nature. Being conscious of this, the doctor is annoyed and humiliated by the never-ceasing slander of the Approved Societies, and by the burdensome system of certificates which they have succeeded in fixing on him. Any such attitude of mind is naturally reflected in a lessened ability to cast aside his own troubles in order that he may thoroughly enter into those of his patient—this is an essential of all successful treatment. Judging from our own experience in the past, any increase of Approved Society control cannot but accentuate the evils it has already caused; and although the doctor may suffer first, the public will suffer last and most.

It is natural that most medical men of to-day state that they will on no account put their sons into the medical profession. It may confidently be expected that the quality of entrants into the profession will fall off, a process accentuated by the many free scholarships at certain Universities. Human nature being what it is, one finds it desirable that the doctor should be by education or social standing superior to his patient. He thus starts with the patient predisposed to respect his opinion and instructions. The lower the general culture of the doctor, the smaller the number of patients to whom he can bring acceptable advice. The Approved Societies are driving away from the profession the best type of man; it is a significant and noteworthy symptom that the medical men of the East End of London are already very largely foreigners, Hindus, Parsees and Negroes. It has been calculated that 25 per cent. of the names on the London panel list are those of foreigners.

The medical profession has always desired a Ministry of Health to co-ordinate the various medical services of the country. At one time it seemed almost as if we had got it, but, at least where National Health Insurance is concerned, the ideal has yet to be realised, and the present Minister is putting it farther and farther away. The ideal is quite simple. It means that there shall be a direct line of responsibility and control between Parliament and the Minister at the head, and the patient and his doctor at the periphery. There should be no intervening body (such as the Approved Societies are) practically uncontrolled and administering at its own sweet will monies collected under the mandamus of the State. And if I am asked what I mean by "practically uncontrolled" I shall be glad to make that plain.

The large political power of the Approved Societies with their enormous vested interest (for the Approved Societies and their parent industrial assurance companies are but loosely divorced) is a direct menace to responsible democratic government in this country. That this power should have already obtained control of taxpayers' contributions (especially under the guise of additional benefits) is very serious. The doctor knows this, and at present he feels it rather more than most, because it is the doctor's scalp which is called for at the moment. Certainly the insured person feels it also, since he is often unable to get his money (which is "saved" by the "careful administration" of the Societies), but since the insured can hardly be expected to understand the intricacies of the Acts, the doctors must speak for them.

At the end of 1923 the Approved Societies come into their own; they begin to exercise the rights which they have purchased by their trust funds. It will be a bad time for the doctor, but the public do well to remember that for every doctor who suffers there must be at least twenty of his patients who suffer with him. The public may also do well to remember that the insistent demand of the profession for an inquiry into the whole working of the Acts has always been refused; the Societies want no inquiry. This circumstance alone should make men think.

Yours, &c.,
GORDON WARD, M.D.,
Vice-President, Medical Practitioners' Union.

### Salisbury and District Infirmary and Hospital League.

*To the Editor of* THE HOSPITAL AND HEALTH REVIEW.

